# Spectral absorption of visual pigments in stomatopod larval photoreceptors

**DOI:** 10.1007/s00359-015-1063-y

**Published:** 2016-01-14

**Authors:** Kathryn D. Feller, Thomas W. Cronin

**Affiliations:** University of Bristol, Life Sciences Building Tyndall Avenue, Bristol, BS8 1TQ UK; University of Maryland Baltimore County, 1000 Hilltop Circle, Baltimore, MD 21250 USA

**Keywords:** Stomatopod, Visual pigment, Larva, Microspectrophotometry, Visual ecology

## Abstract

Larval stomatopod eyes appear to be much simpler versions of adult compound eyes, lacking most of the visual pigment diversity and photoreceptor specializations. Our understanding of the visual pigment diversity of larval stomatopods, however, is based on four species, which severely limits our understanding of stomatopod eye ontogeny. To investigate several poorly understood aspects of stomatopod larval eye function, we tested two hypotheses surrounding the spectral absorption of larval visual pigments. First, we examined a broad range of species to determine if stomatopod larvae generally express a single, spectral class of photoreceptor. Using microspectrophotometry (MSP) on larvae captured in the field, we found data which further support this long-standing hypothesis. MSP was also used to test whether larval species from the same geographical region express visual pigments with similar absorption spectra. Interestingly, despite occupation of the same geographical location, we did not find evidence to support our second hypothesis. Rather, there was significant variation in visual pigment absorption spectra among sympatric species. These data are important to further our understanding of larval photoreceptor spectral diversity, which is beneficial to ongoing investigations into the ontogeny, physiology, and molecular evolution of stomatopod eyes.

**Electronic supplementary material:**

The online version of this article (doi:10.1007/s00359-015-1063-y) contains supplementary material, which is available to authorized users.

## Introduction

Stomatopods are known for the elaborate visual systems found in adults of many species. Adult stomatopod eyes have the largest reported photoreceptor diversity in a single eye, which in some species can include up to 16 classes of photoreceptors (Marshall et al. 1991a). These include specialized receptor classes for detecting a wide spectral range of visible (Cronin and Marshall 1989; Marshall et al. 1991a, b; Cronin et al. 1993; Cronin et al. 1994, Marshall et al. 1996; Chiao et al. 2000a) and ultraviolet (Marshall and Oberwinkler 1999; Bok et al. 2014) light, as well as detecting linearly and circularly polarized light (Marshall et al. 1991a; Marshall et al. 1999; Chiou et al. 2008; for review of stomatopod vision see Cronin et al. 2014). Many of these specialized receptor classes are found in the six rows of often enlarged ommatidia that run along the equator of many adult eyes, referred to as the midband.

By comparison, stomatopod larvae lack the unusual, specialized ocular features of adults—and instead possess compound eyes similar to those of other zooplanktonic crustacean larvae (Cronin et al. 1995; Nilsson 1995). Larval stomatopod eyes are characterized by a uniform arrangement of ommatidial units (with no midband), transparent apposition optics, and a single spectral class of photoreceptor (Cronin et al. 1995; Jutte et al. 1998; Cronin and Jinks 2001). Throughout the larval progression, which depending on the species can include up to 11 pelagic stages, the eyes are understood to maintain this generic anatomy and physiology with only an increase in size, or number of ommatidia, as the animal grows. The stark differences between larval and adult stomatopod eye structures do not become overtly apparent until metamorphosis. In the final hours of the terminal larval stage, the adult retina develops completely separate from, but adjacent to, the preexisting larval eye tissue and persists into the juvenile adult stage until the larval tissue is completely pushed aside and reabsorbed (Williams et al. 1985; Cronin et al. 1995; Feller et al. 2014). This unusual process of eye development may arise from altered physiological demands between larval and adult eye structures (which are presumably adapted to disparate light environments and ecological behaviors), making it necessary to rebuild the eye anew rather than modifying the existing larval structures into the adult form.

Though stomatopod larval eyes are much simpler than those of adults, they are exquisitely adapted for life in the pelagic environment. For example, the transparent apposition optics (provided by the arrangement of the crystalline cones) maximizes the overall transparency of the eye by condensing the opaque retina to a small ball at the center of the organ (Nilsson 1983). This results in an eye that appears much smaller than it actually is, making the animal more difficult to detect by visual predators in the water column (Nilsson 1983; Cronin and Jinks 2001). Additionally, reflecting structures overlying the condensed retina serve to further camouflage the eyes’ appearance (Feller and Cronin 2014).

While there is a rich body of work characterizing the spectral systems of adult stomatopod eyes (for review see Cronin et al. 2014), very little is reported about the spectral sensitivity of larval photoreceptors. Currently, the visual pigment absorption spectra are known for only four species of stomatopod larvae (Table 1; Cronin et al. 1995; Jutte et al. 1998; Cronin and Jinks 2001; Feller et al. 2014). In the present study, we used microspectrophotometry (MSP) to test two hypotheses surrounding larval photoreceptor spectral sensitivity. First, we wished to test whether the presence of a single dominant spectral class of visual pigment is a ubiquitous characteristic of crustacean larval retinas in diverse taxonomic lineages. This hypothesis was founded on the results of MSP studies from four previously investigated stomatopod species (*Gonodactylaceus falcatus,* Cronin et al. 1995; *Pullosquilla thomassini* and *P. littoralis,* Jutte et al. 1998; and *Squilla empusa,* Cronin and Jinks 2001). Since this generalized assumption of larval visual pigments is based on very few species, we wished to further test this hypothesis through expansion of sampling in the stomatopod lineage.

**Table 1:** Summary of average visual pigment absorption maxima known for stomatopod larvae and for the corresponding adults

Species	Larval *λ*_max_ (nm)	Adult *λ*_max_ (nm)
Squilloidea		
***Alima pacifica***	**467**	**479**
*Squilla empusa* ^a^	509^c^	507^c^
***Unknown squilloid***	**449**	–
Lysiosquilloidea		
*Pullosquilla litoralis* ^a^	461^d^	404, 425, 446, 455, 469, 478, 492, 509, 527, 540^d^
*Pullosquilla thomassini* ^a^	447^d^	405, 445, 452, 456, 467, 481, 483, 489, 509^d^
***Pullosquilla thomassini***	**480**	405, 445, 452, 456, 467, 481, 483, 489, 509^d^
***Lysiosquillina maculata***	**501**	397, 416, 434, 461, 492, 499, 500, 501, 516, 517, 538^b, f^
***Unknown lysiosquilloid***	**504**	–
Gonodactyloidea		
*Gonodactylaceus falcatus* ^a^	499^f^	400, 442, 443, 473, 510, 513, 518, 525, 531, 551^e^
***Gonodactylaceus falcatus*** ^a^	**504** ^g^	**532** ^g^
***Gonodactylellus affinis***	**445**	400, 424, 454, 474, 496, 500, 509, 521, 541, 546^d^
***Odontodactylus cultrifer***	**439**	–

Dashes indicate adult stages where no data are available. Bold font denotes species sampled in the present study

*λ*_*max*_ wavelength of peak absorption, *n* number of rhabdoms sampled per species

^a^Denote previously published species

^b^Indicates adult data from *Lysiosquillina sulcata*, a close relative to *L. maculata*

^c^Cronin and Jinks (2001)

^d^Jutte et al. (1998)

^e^Cronin et al. (1995)

^f^Cronin et al. (1993)

^g^Feller et al. (2014)

In addition, we wished to examine the diversity of photoreceptor spectral sensitivity among species occupying the same habitat. Previous microspectrophotometric studies sampled stomatopod larvae procured from different locations; thus, photoreceptor variation among sympatric larvae remains unknown. The taxonomically diverse larvae included in this study (representing the superfamilies Squilloidea, Lysiosquilloidea, and Gonodactyloidea) were captured at the same location, Lizard Island (Queensland, Australia). Given that larvae perform many of the same generic zooplanktonic behaviors (i.e., feeding, vertical migrations, predator avoidance), we hypothesized that sympatric species possess similar visual pigment absorption spectra that are tuned to the pelagic light environment (~480–500 nm peak; McFarland 1986; Chiao et al. 2000b). The data from this study serve to expand our knowledge of larval visual pigment absorption among a broad taxonomic sample of the stomatopod order and improve our understanding of the peculiar ontogeny of vision in these marine crustaceans.

## Materials and methods

### Animals

Stomatopod larvae are prohibitively difficult to culture in the laboratory, thus all individuals measured in this study were captured from the wild. Larvae were collected at night during the months of July and August (2011) near the shore of Lizard Island (Queensland, Australia). Late stage *Alima pacifica* and *Lysiosquillina maculata* were captured at the surface using dip nets and dive lights (AquaSun eLED, 825 lumens). All other larvae were collected on SCUBA at depths ranging from 3 to 15 m using the same dive lights and resealable plastic bags. Larval eyes were processed for microspectrophotometry (MSP) at the Lizard Island Research Station. Non-ocular tissue was fixed in 100 % ethanol and transported to the University of Maryland Baltimore County (UMBC; Baltimore, Maryland, USA) for DNA barcoding to confirm the species identity of each measured individual.

### DNA barcoding

**Fig. 1:**
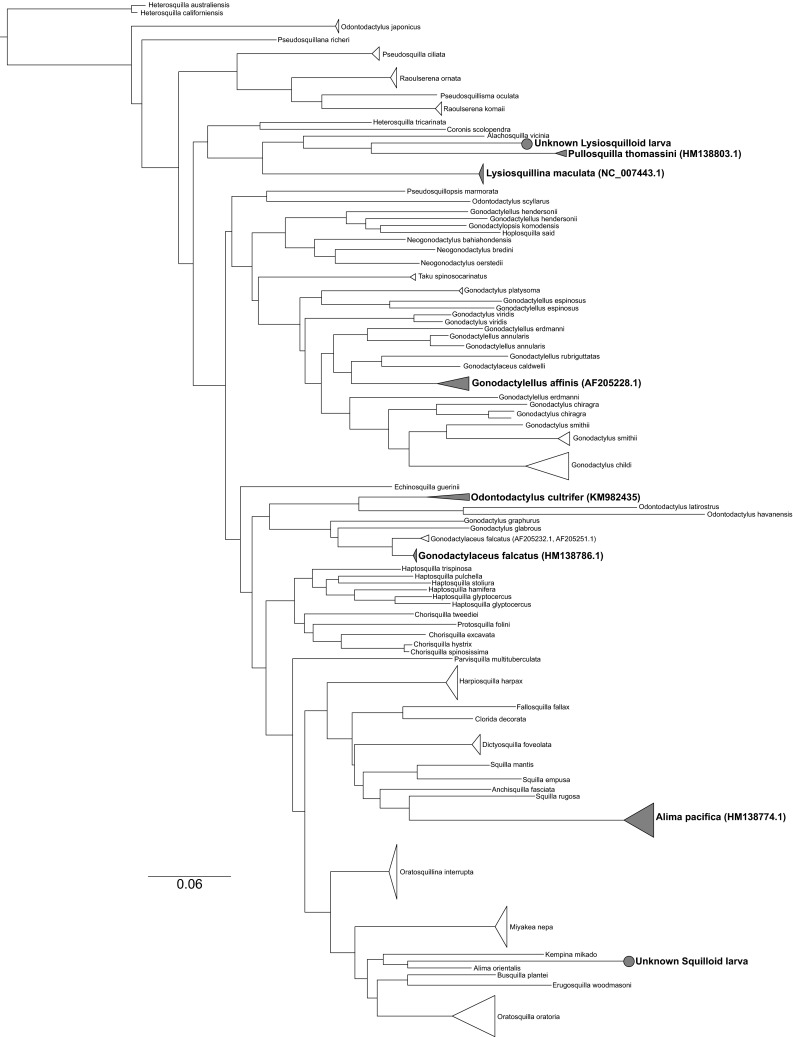
DNA barcoding maximum-likelihood tree constructed from reference (S. Table 1) and sample sequences. *Shaded triangles* represent clades of identified larval sample sequences. Genbank accession numbers for reference sequence of each identified group of larval sample sequences provided in parentheses next to each species name in the figure. Sample sequences included in each shaded clades are as follows: *Alima pacifica* (KM982420, KM982421, KM982422, KM982423, KM982424)*, Gonodactylaceus falcatus* (KM982433)*, Gonodactylellus affinis* (KM982426, KM982428)*, Odontodactylus cultrifer* (KM982427), *Pullosquilla thomassini* (KM982425)*, Lysiosquillina maculata* (KM982431, KM982432, KM982436). The location of the two unknown, individual larval sample sequences (Lysiosquilloid, KM982429; Squilloid, KM982430) are each highlighted with a *shaded circle*. *Scale bar* genetic distance

Fixed, non-ocular tissue from each individual larva measured by MSP was transported to UMBC and stored at 4 °C for up to 1 month before processing. Genomic isolation and DNA sequencing methods as they pertain to stomatopod larval DNA barcoding are published in detail elsewhere (Feller et al. 2013). These methods, in brief, include isolation of genomic DNA from individual specimens and amplification of the cytochrome oxidase I mitochondrial gene (CO1) from each sample using degenerate primers and the polymerase chain reaction. CO1 genes were then sequenced, and species identities were determined through construction of a maximum likelihood phylogeny from 98 reference (S. Table 1) and 16 sample sequences (Fig. 1). According to the phylogenetic relationships established in previous studies (Porter et al. 2010), the phylogeny was rooted using two *Hemisquilla* species: *Hemisquilla australiensis* and *H. californiensis*. Experimental larval CO1 sequences were identified using criteria that consider positive identification of a sample sequence if it is reciprocally monophyletic with a reference sequence or if the sequence divergence between sample and reference is less than 3 % (Barber and Boyce 2006). Identified larval sample sequences were then deposited into GenBank.

### Microspectrophotometry

Prior to experimentation, all animals were dark adapted in a light-tight container within a dark room. Early stage larvae were adapted overnight, whereas late-stage larvae (which were likely to molt into the postlarval form) were adapted for a minimum of 3 h. Under red-light illumination, the eyes were dissected from the larval body and immediately flash-frozen using difluoroethane spray and mounted for cryosectioning. The frozen tissue was mounted in a cryomicrotome at −30 °C and sectioned at thicknesses of 10–12 µm. Each section was lightly fixed in filtered seawater containing 0.5 % glutaraldehyde (to promote photobleaching) and mounted between two coverslips using a ring of silicone grease to contain the mounting solution.

The apparatus used for this research was a field-portable, single-beam, halogen light source microspectrophotometer (described in Loew 1982). A 0.5 neutral density filter (Edmund Optics, UV–VIS 12.5 mm diameter) was used to decrease the light intensity of the beam and maintain the optimal range of high voltage to the photomultiplier tube (−500 to −700 V). A diffuse red LED light (>600 nm) was directed onto a pellicle beam splitter located beneath the microscope stage to provide background illumination of the sample during positioning of the beam. While viewing eye sections under this background illumination, a beam (set to 650 nm) was adjusted to fit within the rhabdom of a single photoreceptive unit. The beam was then moved to the area immediately outside of the tissue section to determine the baseline spectrum. A new baseline spectrum was collected with every adjustment to the beam illumination or diameter, as well as with each new sample preparation. Since photoreceptor morphology is understood to be uniform across stomatopod larval retinas, rhabdoms were sampled one at a time from all regions of the eye. To avoid light contamination between sampled rhabdoms, no two rhabdoms were measured within the same viewable (and thus illuminated) area. For each rhabdom sampled, an initial dark-adapted absorption spectrum was obtained over a wavelength range of 350–750 nm in 1-nm steps driven by a stepper-motor spiral cam monochromator (Loew 1982). The computer-controlled beam was then used to photobleach the rhabdom with full-spectrum (350–750 nm), white light for 3–5 min before collecting a second scan. Neither the beam nor the tissue was moved during this procedure.

Absorption spectra of bleachable photopigments within individual rhabdoms were calculated by taking the difference between the bleach and initial spectra. Each resulting curve was then fit to an A1 visual pigment template (Govardovskii et al. 2000) using a least-squares procedure (described in Cronin et al. 1994) to determine the wavelength of peak absorption of an individual rhabdom (*λ*_max_). Additionally, the average *λ*_max_ for each species was generated by averaging all photobleach data measured from a given species and fitting the resulting curve to a series of A1 template spectra (Govardovskii et al. 2000).

### Statistics

The *λ*_max_ values from individual rhabdoms of a given species were plotted using the boxplot function in the graphics package of R version 2.14 (R Foundation for Statistical Computing, 2012). Using these data, we tested for variation in visual pigment λ_max_ among species using an ANOVA with Scheffe post hoc analysis (*α* = 0.05). ANOVA and post hoc analyses were also performed in R.

## Results

**Fig. 2:**
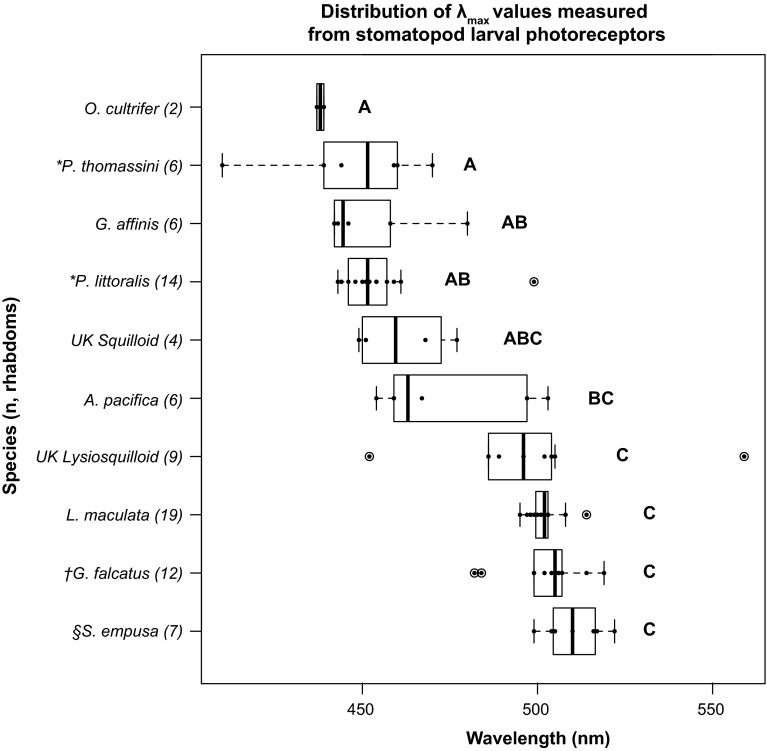
*Box plot* of individual rhabdom *λ*_max_ variation measured from stomatopod larva retinas. *Letters* *A*–*C* denote significance groups from an ANOVA with Scheffe post hoc analysis. *α* = 0.05. *Box* interquartile range, *whiskers* 1.5 interquartile range, *open circles* outliers, *closed circles* individual rhabdom data points, *UK* unknown species. The median is denoted by a *thick*, *black line* drawn horizontally in each *box*. *Scale bar* denotes genetic distance. The number of rhabdoms, *n*, reported from each species are indicated in *parentheses* next to species names. *Asterisk* data from Jutte et al. (1998); *dagger* data from Feller et al. (2014) and Cronin et al. (1995); *section symbol* data from Cronin and Jinks (2001)

**Fig. 3:**
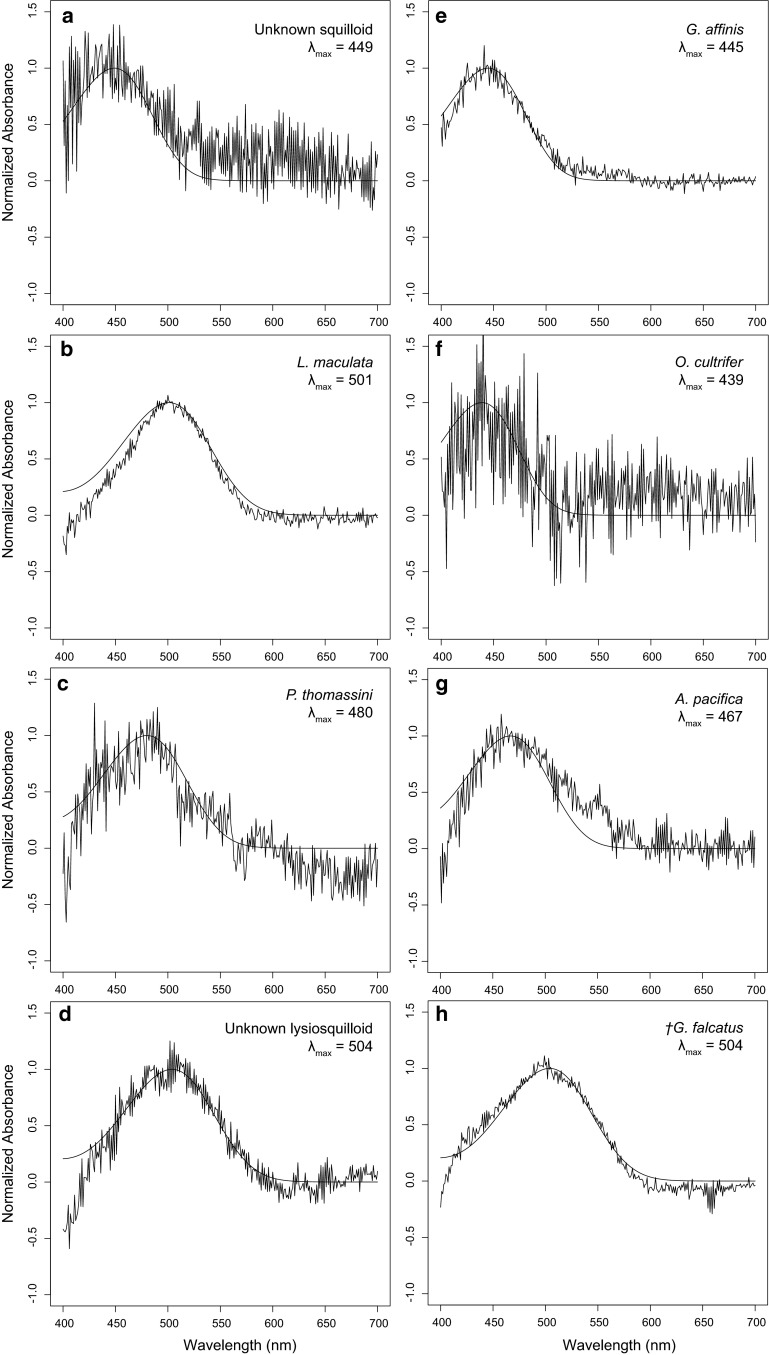
Average visual pigment absorption measurements from larval retinas of eight stomatopod species. Absorption curves calculated from rhabdom data (*n* values reported in Fig. 2) and fit to a template for an A1 chromophore (*smooth line*). *Dagger in h* denotes data published in Feller et al. (2014)

Visual pigment absorption spectra were measured from eight species of stomatopod larvae (Table 1; Figs. 2, 3) whose identities were confirmed via DNA barcoding (Fig. 1). A limitation of performing animal identification post hoc is that the number of individuals sampled per species in the primary experiment (MSP) cannot be controlled. As a result, the final data-set includes absorption measurements from multiple individuals of some species and single individuals for others. These species and the number of individuals sampled (in parentheses) from each species are as follows: *Alima pacifica* (5), *Gonodactylaceus falcatus* (1), *Gonodactylellus affinis* (2), *Odontodactylus cultrifer* (1), *Pullosquilla thomassini* (1)*, Lysiosquillina maculata* (3), an unknown lysiosquilloid (1), and an unknown squilloid species (1) (S. Table 2; Fig. 1). The individual *O. cultrifer* larva fell outside of our 3 % sequence divergence minimum criteria for species assignment with a divergence of 4.7 % from the reference sequence (S. Table 2). However, we were able to assign this individual to species given its relative distance from other *Odontodactylus* species and reciprocal monophyly with the *O. cultrifer* reference (Fig. 1). The unknown larvae also presented genetic distances outside of our minimum criteria, but failed to present sufficient reciprocal monophyly and were thus assigned to superfamily as the lowest taxonomic unit (S. Table 2; Fig. 1). Thus, the nearest reference to the unknown lysiosquilloid larva was *P. thomassini* (Lysiosquilloidea; 16.3 % genetic distance, S. Table 2) and the unknown squilloid was sister to the *A. pacific* clade (Squilloidea; 18 % genetic distance, S. Table 2).

Though the present study provides data from a single rhabdom of an early-stage *P. thomassini* larva that agrees with previously published results on this species, these data were insufficient for statistical analysis (Table 1; Fig. 3). To further analyze *λ*_max_ variation among stomatopod larval species, we included data previously published for species both collected (*G. falcatus,* Feller et al. 2014) and not collected (*Pullosquilla thomassini* and *P. littoralis;* Jutte et al. 1998; and *Squilla empusa*; Cronin and Jinks 2001) from the Lizard Island reef platform. Note that though *P. thomassini* and *P. littoralis* species do occur at Lizard Island, the previously published data from these species were generated from laboratory-reared larvae hatched from adult specimens captured in Moorea (French Polynesea; Jutte et al. 1998). An ANOVA test with Scheffe post hoc analysis (*p* < 0.001) reveals two significantly different groups of visual pigment classes: a short wavelength group (*O. cultrifer, P. thomassini, G. affinis, P. litoralis*) and a long wavelength group (Unknown lysiosquilloid*, L. maculata, G. falcatus, S. empusa*). The visual pigments of two species, *A. pacifica* and the unknown squilloid, were intermediate to the short- and long-wavelength significance groups (Fig. 2).

## Discussion

In accordance with previous findings (Cronin et al. 1995; Jutte et al. 1998; Cronin and Jinks 2001), the data presented in this study support the hypothesis that a single spectral class of photoreceptor is expressed in larval stomatopod eyes (Table 1). However, the data did not support our second hypothesis that stomatopod larvae from the same habitat possess photoreceptors with similar absorption spectra. Though all sampled individuals occur in the same pelagic habitat, near Lizard Island (Queensland, Australia), absorption spectra maxima (or *λ*_max_ values) varied significantly among species (Fig. 2). Detailed discussions of each of these findings are provided below.

Using MSP, we characterized photoreceptor spectral absorption in eight sympatric species of stomatopod larvae, six of which were previously uncharacterized (Table 1; Figs. 2, 3). The three superfamilies represented by these species make up ~87 % of the extant stomatopod biodiversity (*Squilloidea, Lysiosquilloidea, and Gonodactyloidea*; Porter et al. 2010), presenting a broad taxonomic sampling of the stomatopod lineage. Only a single spectral class of photoreceptor was found in retinas of each sampled species, which further supports the understanding that a single spectral class of visual pigment is a shared trait among stomatopod larval retinas. Though ultraviolet (UV)-sensitive, R8 photoreceptors are suggested to exist in the retinas of marine crustacean larvae (Douglass and Forward 1989), neither MSP evidence of a UV-absorbing visual pigment nor anatomical evidence of R8 cell structures have been found in stomatopod larvae (Feller pers. observ.). Given the current data, we cannot reject the hypothesis that only a single, spectral class of visual pigment is expressed in stomatopod larval retinas at any given developmental time point. However, we strongly suggest further investigation into the photoreceptor spectral absorption of *A. pacifica* and other squilloid larvae that present a broad, though not statistically different, range of *λ*_max_ values (Fig. 2). Additionally, the presence of outliers in many of the sampled species may represent a small population of photoreceptors of a different spectral class (Fig. 2). This question can be adequately addressed by additional techniques, such as molecular characterization of larval opsin genes and their expression patterns across the retina and/or by intracellular recordings of photoreceptor cells.

Changes in visual pigment expression at metamorphosis remain an ongoing question in the literature (Cronin et al. 1995; Cronin and Jinks 2001). Prior to this investigation, data from several stomatopod studies suggested expression of the larval visual pigment in the adult retina (Table 1; *P. thomassini* and *P. litoralis;* Jutte et al. 1998; *S. empusa,* Cronin and Jinks 2001) while others suggested that the larval pigment is lost at metamorphosis (Table 1; *G. falcatus,* Cronin et al. 1995). Our data serve to expand the number of species for which we have larval and adult visual pigment absorption data; however, they do not resolve this question. While the squilloid and lysiosquilloid larvae demonstrate expression of a similar visual pigment class in larval and adult retinas (*A. pacifica, S. empusa, P. thomassini, P. litoralis,* and *L. maculata*), data from the gonodactyloid species (*G. falcatus* and *G. affinis*) suggest that the larval visual pigment is distinct from any visual pigments found in the adult eye (Table 1). A characterization of opsin gene (or visual pigment protein) expression throughout ontogeny of individual species from larvae to adults will serve to answer this question.

One of the most unexpected findings of this study was the amount of variation in absorption spectra among different species of stomatopod larvae known to occur in the same pelagic habitat (Fig. 2). The pelagic environment is a featureless world in which all larvae must perform similar visual tasks for feeding and survival. We predicted that larval species from the same environment, the waters near Lizard Island, would express similar absorption spectra in their photoreceptors that is typical of the light environment in which they are found (maximum irradiance near 480–500 nm; McFarland 1986; Chiao et al. 2000b; see data in Cronin 2006). Though some species matched our prediction, visual pigments in three of the sampled species maximally absorbed at wavelengths significantly shorter than those of their sympatric heterospecifics, leading us to reject our initial hypothesis that larval species in the same habitat share similar visual pigment absorption spectra. Since larval retinas are independent of the adult tissue (per the double-retina eye development described in the introduction), these differences can neither be attributed to differences in adult retinal sensitivity, nor to the evolutionary relationships of the sampled species. Thus, we must posit alternate hypotheses to account for the observed differences in larval photoreceptor diversity.

Variations in spectral tuning among species, whether by the expression of different light-absorbing visual pigments or by the interaction with other optical structures (such as screening pigments or filters; Marshall et al. 1991a, b; Jutte et al. 1998), are typically driven by differences in light environment or in animal ecology (Cronin et al. 1993; Chiao et al. 2000a, b). Since the larvae sampled in this study were all understood to occur in the same light environment, differences in the ecological niches occupied by each species may be responsible for driving the expression of significantly different photoreceptor classes among species (Fig. 2). Specialized behavioral tasks such as vertical migration, feeding, or adult settlement cues may also contribute to the observed differences in spectral sensitivity. Differences in vertical migratory behavior in particular may result in selection towards different photoreceptor spectral sensitivities, since the light environment changes substantially with depth (McFarland 1986). Until more is known regarding stomatopod larval ecology and behavior, the adaptive significance of different photoreceptor classes in each species will remain unknown.

Though differences in ecology offer a potential explanation for the unexpected variation in the *λ*_max_ values of sympatric species, an alternate hypothesis that considers the developmental stages of sampled individuals may also explain these results. All species in the short-wavelength sensitive group were sampled in the earliest stages of their larval progression. Since the larval stages are not described for most species, we cannot ascertain the exact stage of these individuals; however, it appears likely that these wild-caught larvae were in their first, or very close to first, zoeal stage of development. All individuals in the long-wavelength group were sampled at a later or last stage of the larval developmental phase. These individuals included terminal stage larvae (*A. pacifica* and *L. maculata*), newly molted postlarvae with large double retinas (*G. falcatus*), and a midstage larva (unknown lysiosquilloid, assessed on general size and appendage development). The data included from previously published studies of allopatric larvae also follow this pattern. *S. empusa* visual pigments from late stage larvae (Cronin and Jinks 2001) absorb significantly longer wavelengths than those of either *P. thomassini* or *P. litoralis* larvae, which were sampled in their first stage after hatching in the laboratory (Jutte et al. 1998). In general, it appears common for late-stage larvae to express a set of visual pigments absorbing at longer wavelengths than those of early-stage larvae from the same species.

It has been reported that larval behavior, specifically maximum depth range for vertical migration, changes as stomatopods progress from early to late stages of larval development (Dingle 1968; Ohtomi et al. 2005). Thus, in accordance with such ontogenetic changes in behavior we hypothesize that stomatopod larvae undergo an ontogenetic shift in photoreceptor spectral sensitivity during the larval phase of life, whereby early stage larvae express short-wavelength sensitive visual pigments and the visual pigments of later larval stages are long-wavelength shifted. Characterization of larval visual pigment absorption spectra or opsin gene expression patterns during each stage of development (from embryo to postlarva) of a single species will serve to test this hypothesis directly.

This body of work contributes to our understanding of stomatopod larval visual ecology and provides a foundation on which to build further studies of this system. Many of the questions raised by this study can be addressed through characterization of the opsin proteins expressed throughout the visual ontogeny of stomatopod crustaceans. The further pursuit of stomatopod larval visual research will serve to bring additional elements into the greater story of stomatopod eye evolution.

## Electronic supplementary material


**S. Table 1**. Reference cytochrome oxidase subunit I sequences used to construct maximum likelihood tree for DNA barcoding identification. With the exception of *O*. *cultrifer*, all sequences were obtained from GenBank. The reference sequence for *O*. *cultrifer* is a new submission with this work. (DOCX 106 kb)

**S. Table 2**. Cytochrome oxidase subunit I percent similarities (genetic distance) between larval (left column) and reference sequences (top row). Reference sequence accession numbers listed with each species code. Highest percent similarity values highlighted in bold. In cases where multiple references exist for a given species, only the sequence with the highest percent similarity is reported. Species codes: *Lm*, *Lysiosquillina maculata*; UK Sq, unknown squilloid; *Ap*, *Alima pacifica*; *Pt*, *Pullosquilla thomassini*; UK Ly, unknown lysiosquilloid; *Ga*, *Gonodactylellus affinis*; *Gf*, *Gonodactylaceus falcatus*; *Oc*, *Odontodactylus cultrifer*. Lar, larva (DOCX 77 kb)
