# Supplementary material for: Spectral absorption of visual pigments in stomatopod larval photoreceptors
Source: J Comp Physiol A Neuroethol Sens Neural Behav Physiol. 2016 Jan 14;202:215–23. doi: 10.1007/s00359-015-1063-y (PMC4759216; doi:10.1007/s00359-015-1063-y)
Supplement: Supplementary file 1 — S. Table 1. Reference cytochrome oxidase subunit I sequences used to construct maximum likelihood tree for DNA barcoding identification. With the exception of O. cultrifer, all sequences were obtained from GenBank. The reference sequence for O. cultrifer is a new submission with this work. (DOCX 106 kb) [file 359_2015_1063_MOESM1_ESM.docx]

Supplemental Table 1.

| **Species** | **Genbank Accession Number(s)** |
| --- | --- |
| Squilloidea |  |
| *Alima orientalis* | HM138773.1 |
| *Alima pacifica* | HM138774.1 |
| *Anchisquilla fasciata* | FJ229760.1 |
| *Busquilla plantei* | HM138775.1 |
| *Clorida decorata* | FJ229763.1, FJ229762.1, FJ229762.1 |
| *Dictyosquilla foveolata* | FJ229764.1, FJ229765.1, FJ229766.1, FJ229767.1 |
| *Erugosquilla woodmasoni* | FJ229768.1, FJ229769.1 |
| *Fallosquilla fallax* | HM138781.1 |
| *Kempella mikado* | HM138792.1 |
| *Miyakella nepa* | FJ229780.1, FJ229781.1 |
| *Oratosquillina interrupta* | FJ229790.1, FJ229795.1 |
| *Oratosquilla oratoria* | FJ229782.1, FJ229783.1, FJ229784.1, FJ229785.1, FJ229786.1, FJ229787.1 |
| *Squilla empusa* | HM138809.1, NC_007444.1 |
| *Squilla mantis* | GQ328967.1, JQ624005.1, KC311428.1 |
| *Squilla rugosa* | HM138810.1 |
| Gonodactylloidea |  |
| *Chorisquilla excavata* | HM138776.1 |
| *Chorisquilla hystrix* | HM138777.1 |
| *Chorisquilla twediei* | HM138778.1 |
| *Echinosquilla guerinii* | HM138780.1 |
| *Gonodactylaceus caldwelli* | DQ440594.1 |
| *Gonodactylaceus falcatus* | HM138786.1, AF205230.1, AF205232.1, AF205251.1 |
| *Gonodactylellus affinis* | AF205228.1 |
| *Gonodactylellus annularis* | HM138783.1, AF205226.1 |
| *Gonodactylellus espinosus* | HM138782.1 |
| *Gonodactylellus erdmanni* | DQ440593.1, GQ260981.1 |
| *Gonodactylellus hendersonii* | AF205225.1, AF205231.1 |
| *Gonodactylus childi* | HM138784.1, AF205227.1, AF205229.1, AF205246.1, AF205249.1 |
| *Gonodactylus chiragra* | HM138785.1, NC_007442.1 |
| *Gonodactylus platysoma* | HM138787.1 |
| *Gonodactylus smithii* | HM138788.1 |
| *Gonodactylus viridis* | AF205224.1 |
| *Hemisquilla californiensis* | HM138791.1 |
| *Hemisquilla australiensis* | AF205252.1 |
| *Haptosquilla glyptocercus* | AF205239.1, HM138789.1 |
| *Harpiosquilla harpax* | FJ229770.1, FJ229771.1, FJ229772.1, FJ229773.1, FJ229774.1 |
| *Haptosquilla trispinosa* | HM138790.1 |
| *Neogonodactylus bahiahondensis* | HM138794.1 |
| *Neogonodactylus bredini* | HM138795.1 |
| *Neogonodactylus oerstedii* | HM138796.1 |
| *Odontodactylus japonicus* | FJ229797.1, FJ229798.1 |
| *Odontodactylus latirostris* | HM138797.1 |
| *Odontodactylus cultrifer* | KM982435 |
| *Odontodactylus scyllarus* | HM138798.1 |
| *Protosquilla folini* | HM138799.1 |
| *Pseudosquilla ciliata* | HM138800.1 |
| *Pseudosquillana richeri* | HM138802.1 |
| *Raoulserena hieroglyphica* | HM138805.1 |
| *Raoulserena komaii* | HM138804.1 |
| *Raoulserena ornata* | HM138806.1 |
| *Raoulserena oxyrhyncha* | HM138807.1 |
| *Raoulserena pygmaea* | HM138808.1 |
| *Taku spinosocarinatus* | HM138811.1 |
| Lysiosquilloidea |  |
| *Coronis scolopendra* | HM138779.1 |
| *Lysiosquillina maculata* | NC_007443.1 |
| *Pullosquilla thomassini* | HM138803.1 |
| Parasquilloidea |  |
| *Pseudosquillopsis marmorata* | HM138801.1 |
