# Supplementary material for: Spectral absorption of visual pigments in stomatopod larval photoreceptors
Source: J Comp Physiol A Neuroethol Sens Neural Behav Physiol. 2016 Jan 14;202:215–23. doi: 10.1007/s00359-015-1063-y (PMC4759216; doi:10.1007/s00359-015-1063-y)
Supplement: Supplementary file 2 — S. Table 2. Cytochrome oxidase subunit I percent similarities (genetic distance) between larval (left column) and reference sequences (top row). Reference sequence accession numbers listed with each species code. Highest percent similarity values highlighted in bold. In cases where multiple references exist for a given species, only the sequence with the highest percent similarity is reported. Species codes: Lm, Lysiosquillina maculata; UK Sq, unknown squilloid; Ap, Alima pacifica; Pt, Pullosquilla thomassini; UK Ly, unknown lysiosquilloid; Ga, Gonodactylellus affinis; Gf, Gonodactylaceus falcatus; Oc, Odontodactylus cultrifer. Lar, larva (DOCX 77 kb) [file 359_2015_1063_MOESM2_ESM.docx]

Supplemental Table 2.

|  | ***Ap* HM138774.1** | ***Pt* HM138803.1** | ***Lm* NC_007443.1** | ***Ga* AF205228.1** | ***Gf* HM138786.1** | ***Oc* KM982435** |
| --- | --- | --- | --- | --- | --- | --- |
| ***Lm* lar1 KM982431** | 77 | 78.3 | **99.4** | 80.5 | 78.5 | 79.7 |
| ***Lm* lar2 KM982432** | 77.2 | 78.8 | **99.5** | 80.9 | 78.5 | 79.8 |
| ***Lm* lar3 KM982436** | 77.5 | 78.6 | **99.5** | 80.7 | 78.8 | 79.6 |
| **UK Sq KM982430** | **81.1** | 79.6 | 80.6 | 80.1 | 80.2 | 80.2 |
| ***Ap* lar1 KM982420** | **98.1** | 78.5 | 79.2 | 79.7 | 81.4 | 79.9 |
| ***Ap* lar2 KM982421** | **98.4** | 78.6 | 79.2 | 79.9 | 81 | 80.4 |
| ***Ap* lar3 KM982423** | **98** | 78.5 | 78.9 | 80.2 | 81 | 80.3 |
| ***Ap* lar4 KM982422** | **98.4** | 78.6 | 79.1 | 80.2 | 81.3 | 80.4 |
| ***Ap* lar5 KM982424** | **99.5** | 78.1 | 78.7 | 79.4 | 81.1 | 79.7 |
| ***Pt* lar1 KM982425** | 79.1 | **99** | 79.6 | 79.9 | 82.5 | 82.1 |
| **UK Ly KM982429** | 78.3 | **83.7** | 81.5 | 77.2 | 82.4 | 81.1 |
| ***Ga* lar1 KM982428** | 80.7 | 81 | 80.9 | **96.3** | 84.6 | 83.3 |
| ***Ga* lar2 KM982426** | 79.9 | 81.1 | 81.5 | **96.2** | 84.3 | 83.2 |
| ***Gf* lar1 KM982433** | 81.4 | 82.7 | 79.6 | 83.9 | **99.8** | 84.7 |
| ***Oc* lar1 KM982427** | 80.2 | 80.8 | 79.7 | 82.8 | 84.7 | **95.3** |
